# Supplementary material for: The Use of Selected Ion Flow Tube-Mass Spectrometry Technology to Identify Breath Volatile Organic Compounds for the Detection of Head and Neck Squamous Cell Carcinoma: A Pilot Study
Source: Medicina (Kaunas). 2019 Jun 25;55(6):306. doi: 10.3390/medicina55060306 (PMC6631766; doi:10.3390/medicina55060306)
Supplement: Supplementary file 1 [file medicina-55-00306-s001.pdf]

Table 1. Median Values of all Volatile Organic Compounds.

Volatile Organic Compounds	Median	
	Healthy	Cancer
1,6-dihydrocarveol	1.94	1.86
2-propanol	31	34.8
3-methylhexane	11.35	11.6
isobutanoic acid	3.065	2.92
isopropylamine	1.01	1.19
methanol	14.5	11.2
p-xylene	1.06	1.14
1-octene	4.635	4.94
1-propanol	31.95	36.3
acetophenone	0.917	0.65
benzoic acid	0.609	0.63
cyclohexanone	4.01	4.17
dodecane	1.63	1.71
isopentane	5.52	5.31
methyl n-propyl sulfide	2.88	2.9
pentanal	2.99	2.59
2-methoxy-2-methyl butane	4.26	4.12
3-hexanone	3.75	3.27
Freon 113	3.01	2.8
acetoin	1.975	2.11
formaldehyde	6	8.08
furan	20.5	23.1
hydrogen cyanide	1.115	2.52
isooctane	4.25	4.17
toluene	0.7735	0.79
1,2,4-trimethylbenzene	1.03	1
camphor	0.957	0.8
dimethyl disulfide	1.05	0.93
ethane	0.7415	0.69
hexanal	4.77	4.84
pentane	13.45	16
3-methylhexane	11.35	11.5
benzene	2.435	3.35
methylcyclopentane	1.31	1.5
propylbenzene	1.09	1.15
tridecane	1.6	1.67
acetone	90.65	112
decane	3.305	3.33
heptanal	2.265	2.23
isoprene	30.9	36
styrene	1.6	0.77
2-methylpentane	9.17	9.62
4-methyloctanoic acid	1.8	1.71
N2 isopentane	7.965	7.73
N2 m-xylene	1.04	1.14
decanal	1.875	1.47
methyl cyclohexane	1.42	1.44
methyl isobutyl ketone	4.535	4.67
methylcyclopentane	1.31	1.5
nonanal	3.645	2.86
1,3-butadiene	0.2165	0.3
1,4-benzoquinone	1.22	1.09

N2 1,1-dichoroethane	4.635	4.56
N2 2-methylundecanal	2.105	2.02
acetophenone	0.911	0.6
butanone	2.365	1.96
cyclopropane	70	88.6
furfural	0.612	0.72
longifolene	0.7215	0.76
octanal	3.12	2.58
1,4-diaminobutane	2.27	2.67
3-methylbutanoic acid	1.975	2.16
ammonia	31.2	33.9
butanoic acid	2.77	2.98
dimethyl sulfide	1.695	1.73
hydrogen sulfide	4.365	4.51
indole	0.3005	0.35
methyl mercaptan	1.135	1.83
1,5-diaminopentane	1.0065	1.25
3-methylindole	0.335	0.35
trimethylamine	9.48	12.8
N2 butanoic acid	2.28	2.7
acetic acid	15.8	17.05
formic acid	20.8	24.3
pentanoic acid	2.27	2.545
propanoic acid	2.09	2.54
1-octene	4.635	4.94
2,2-dimethyl propanoic acid	2.485	2.32
2,3-butanediol	3.89	4.57
acrylonitrile	0.3035	0.33
butanone	2.375	1.96
ethanol	16.5	19.1
limonene	0.9975	0.88
undecane	3.06	2.48
